# Application of the fragment molecular orbital method to discover novel natural products for prion disease

**DOI:** 10.1038/s41598-018-31080-7

**Published:** 2018-08-30

**Authors:** Jiwon Choi, Hyo-Jin Kim, Xuemei Jin, Hocheol Lim, Songmi Kim, In-Soon Roh, Hae-Eun Kang, Kyoung Tai No, Hyun-Joo Sohn

**Affiliations:** 1 Bioinformatics and Molecular Design Research Center (BMDRC), Yonsei University, Seoul, 03722 Korea; 2 OIE Reference Laboratory for CWD, Foreign Animal Disease Research Division, Animal and Plant Quarantine Agency, Gimcheon, Gyeongsangbukdo, 39660 Korea; 3 Department of Biotechnology, Yonsei University, Seoul, 03722 Korea

## Abstract

Conformational conversion of the normal cellular isoform of the prion protein PrP^C^ into an infectious isoform PrP^Sc^ causes pathogenesis in prion diseases. To date, numerous antiprion compounds have been developed to block this conversion and to detect the molecular mechanisms of prion inhibition using several computational studies. Thus far, no suitable drug has been identified for clinical use. For these reasons, more accurate and predictive approaches to identify novel compounds with antiprion effects are required. Here, we have applied an *in silico* approach that integrates our previously described pharmacophore model and fragment molecular orbital (FMO) calculations, enabling the *ab initio* calculation of protein-ligand complexes. The FMO-based virtual screening suggested that two natural products with antiprion activity exhibited good binding interactions, with hotspot residues within the PrP^C^ binding site, and effectively reduced PrP^Sc^ levels in a standard scrapie cell assay. Overall, the outcome of this study will be used as a promising strategy to discover antiprion compounds. Furthermore, the SAR-by-FMO approach can provide extremely powerful tools in quickly establishing virtual SAR to prioritise compounds for synthesis in further studies.

## Introduction

Prions are the infectious agents that cause prion diseases or transmissible spongiform encephalopathies (TSEs), which are neurodegenerative diseases that comprise Creutzfeldt-Jakob diseases (CJD), kuru, fatal familial insomnia, scrapie, and bovine spongiform encephalopathy, affecting both humans and a variety of mammalians^1–3^. These diseases are caused by the conformational change of the normal cellular prion protein (PrP^C^) into a pathogenic conformation (PrP^Sc^), and the accumulation of PrP^Sc^ in the central nervous system^4–6^. PrP^C^ is a ubiquitous glycosylphosphatidylinositol (GPI)-linked glycoprotein that contains a flexible, unstructured N-terminal domain, and a globular C-terminal domain containing two short native antiparallel β strands (β1 and β2) and three α helices (α1, α2, and α3), which are highly conserved among mammalian species^7,8^. PrP^Sc^ is a polymeric and β sheet-enriched isoform, and also possesses abnormal physiochemical properties to drive the progression of prion diseases^9^. Therefore, stabilisation of the native PrP^C^ conformation, preventing the conversion from PrP^C^ to PrP^Sc^, or enhancing the PrP^Sc^ clearance, may reduce the PrP^C^ misfolding rate, and further provide a promising strategy to prevent or cure prion diseases^10^. The origin of BSE has not been fully identified; however, meat and bone meal derived from cattle with BSE are regarded as a major route of BSE transmission to human vCJD. Moreover, the agent causing BSE may be identical to the agent responsible for CJD^11^.

There are only 1.5–2 cases of human-affected prion diseases reported annually worldwide; however, they are invariably fatal, and currently no effective treatments exist^12^. Although the detailed structure of PrP^Sc^ is still unclear, the structure of PrP^C^ has been elucidated by NMR and X-ray diffraction measurements. Therefore, PrP^C^ is the only possible target for rational drug design at this stage^13,14^. Consequently, a variety of antiprion compounds, including pentosane polysulfate, polyamines, amantadine, astemizole, dextran sulphate, Congo red, suramin, rapamycin, and quinacrine have been reported to specifically or non-specifically bind to PrP^C^, acting as chemical chaperones to reduce PrP^Sc^ accumulation in cell culture models of prion diseases^12,14–16^. However, most of the compounds active *in vitro* have failed *in vivo*, and therefore more effective and rational drug discovery strategies are still needed to successfully screen small-molecular chemical libraries^17^. Among the diverse group of antiprion compounds, natural products have well-established safety profiles; thus, they may serve as promising candidates for the treatment of prion diseases^18,19^.

Many studies have reported some antiprion compounds that specifically bind to the hotspot region of PrP^C^^2,15,20^. This hotspot was first identified by the group of Kuwata *et al*.^21^, who proposed one compound, named GN8, which strongly stabilises normal conformation by binding to PrP^C^. Based on several computational studies, the specific binding site of PrP^C^ can be specified as Asn159, Val189, Thr192, Lys194, and Glu196^15,22,23^. Therefore, focusing on the hotspot pocket as the interaction site between GN8 and PrP^C^ may be a promising way to develop new antiprion drugs in the future.

Based on our previous study^24^, a new 3D pharmacophore model was generated and modified using the fragment molecular orbital (FMO)^25^ method to discover novel compounds that can stabilise PrP^C^ and reduce PrP^Sc^ accumulation. The FMO method offers faster computational speeds than the traditional quantum-mechanical (QM) method^26^, and provides accurate information for investigating the chemical nature and binding characteristics of protein-ligand interactions^27^. For this reason, the FMO method was used to analyse the interaction energies between GN8 and PrP^C^ to identify important pharmacophore features for virtual screening of our in-house natural product database. Next, we employed molecular docking and a classical bovine spongiform encephalopathy (BSE)-infected cell-based assay system^28^ to discover two potent natural products, BNP-03 and BNP-08, which showed inhibitory effects by reducing PrP^Sc^ signals in a standard scrapie cell assay (SSCA)^29^.

In this study, we performed molecular dynamics (MD) simulations and additional FMO calculations to validate the molecular interactions between PrP^C^ and natural products (BNP-03 and BNP-08). We found that these two natural products bound to a well-defined hotspot binding site of PrP^C^. We explored key residues that are important for their binding affinity by using pair interaction energies (PIEs) and pair interaction energy decomposition analysis (PIEDA)^30,31^. The PIEs provide a comprehensive list of interactions between ligand and protein to identify the important residues for ligand binding^31^. The PIEDA is a sum of four energy terms: electrostatic, exchange repulsion, charge transfer, and dispersion^32^. The electrostatic and charge transfer are important in salt-bridges, hydrogen bonds, and polar interactions, whereas the dispersion term is referred to as hydrophobic, and the exchange repulsion term describes the steric repulsion between electrons^33^. This information is essential for optimisation and modification of lead compounds to increase the binding affinities of protein-ligand interactions^34^. Overall, the integration of the FMO method with modelling simulations can provide a powerful tool for the validation of drug discovery programmes, and furthermore, it can be highly beneficial for the structural optimisation of compounds in future studies.

## Results

### Application of FMO method for virtual screening

**Figure 1:**
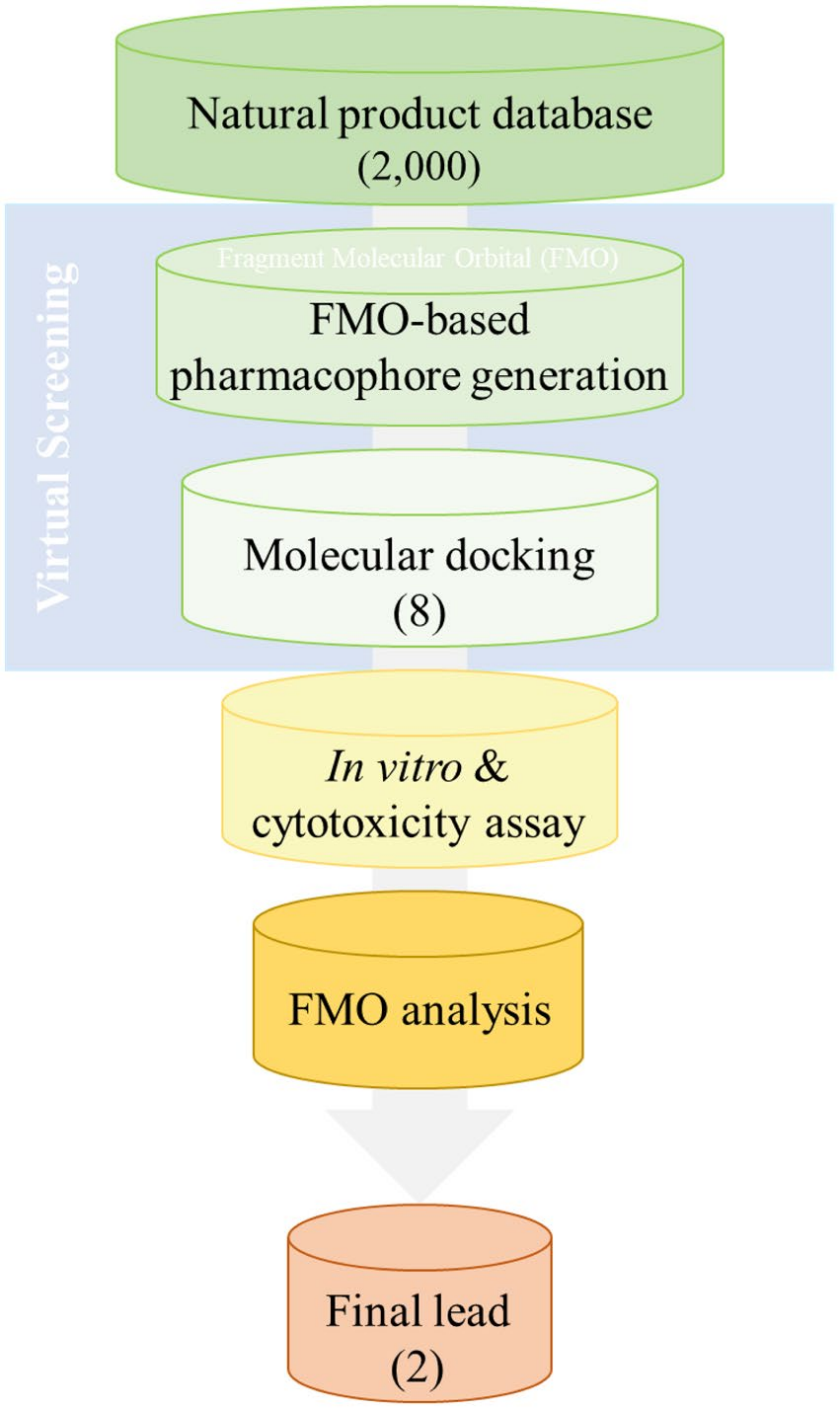
Work flow of FMO-based virtual screening of antiprion drug discovery.

**Figure 2:**
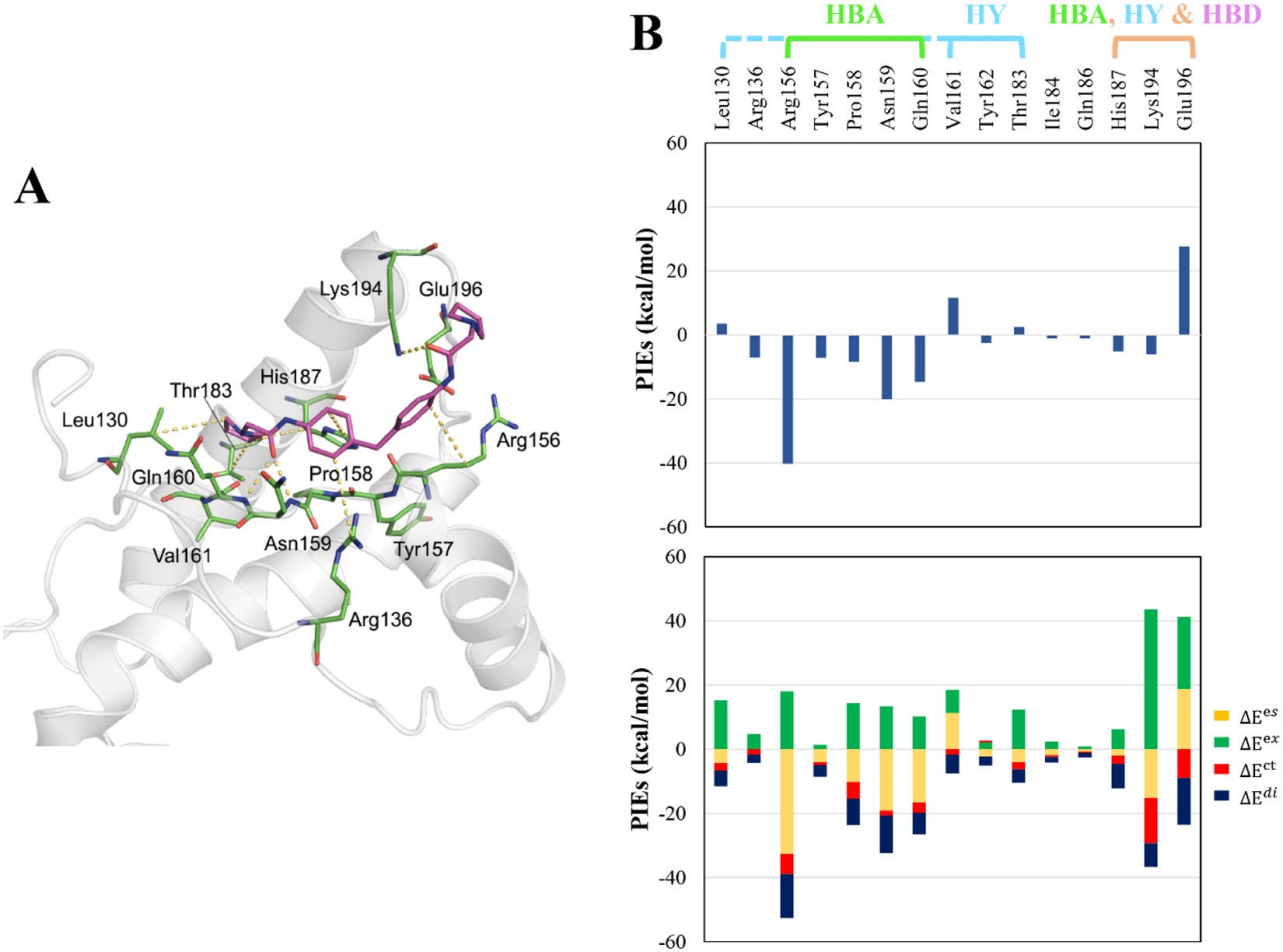
The FMO result for GN8 in complex with PrP^C^. **(A)** The structure of GN8 binding to the hotspot binding pocket of PrP^C^. GN8 is shown in pink, and the residues of the protein are coloured green. Nitrogen and oxygen atoms are coloured in blue and red, respectively. The key interactions according to FMO calculations are marked as yellow dashed lines. **(B)** The top-hand bar plot describes the PIEs of the significant residues in the binding pocket of PrP^C^, and the bottom-hand bar plot describes the PIEDA of these key interactions. The electrostatic, exchange repulsion, charge transfer and dispersion terms are coloured in yellow, green, red, and dark blue, respectively. The residues contribute to generate hydrogen bond acceptor (HBA, green), hydrogen bond donor (HBD, magenta), and hydrophobic (HY, cyan) features.

**Figure 3:**
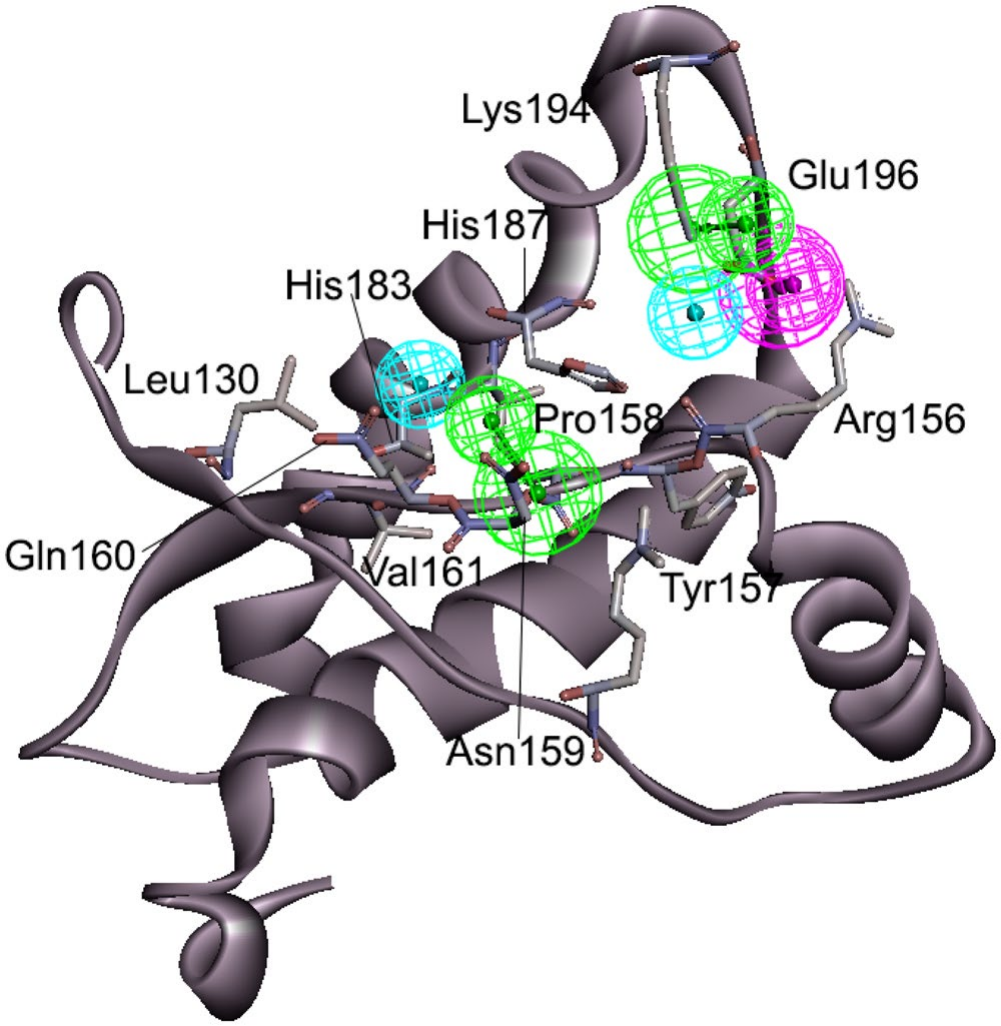
A 3D pharmacophore model of PrP^C^ modified by the FMO method. The model consists of five pharmacophore features, including two HBAs (green), one HBD (magenta), and two HYs (cyan). HBA corresponds to Tyr157, Pro158, Asn159, and Lys194; HBD corresponds to Arg156, and Glu196; HY corresponds to Leu130, Val161, Thr183, and His187.

The flowchart of the discovery process of antiprion compounds performed in the present study is shown in Fig. 1. To develop a novel antiprion agent for the treatment of prion disease, we generated a modified pharmacophore model using the FMO calculation of the docked PrP^C^-GN8 complex^24^. GN8, a known antiprion compound, was well-docked into the hotspot binding site of PrP^C^ with key residues, including Asn159, Gln160, Lys194, and Glu196, which were examined by the FMO calculations performed by Ishikawa *et al*.^35^. To enhance the predictability of the selection of more potent antiprion compounds in drug discovery, we also performed the FMO calculations based on our previous docked PrP^C^-GN8 complex, and detected eight strongly attractive interactions between GN8 and PrP^C^ with eight residues: Arg136, Arg156, Tyr157, Pro158, Asn159, Gln160, His187, and Lys194; these are depicted in Fig. 2 and Supplementary Table S1. These results are in agreement with those of Ishikawa *et al*.^35^, demonstrating the central role of Asn159, Gln160, and Lys194 in the binding affinities of GN8 to PrP^C^. The FMO method also detected three strong repulsive interactions between GN8 and Leu130, Val161, and Glu196 in the binding pocket of PrP^C^. However, the dispersion energies of Leu130 (−4.86 kcal/mol), Val161 (−5.85 kcal/mol), and Glu196 (−14.64 kcal/mol) mainly contribute to form the hydrophobic interactions in the binding pocket of PrP^C^ (Supplementary Table S1). Based on the structural information from the FMO results, we proposed a modified pharmacophore model of PrP^C^ with five important pharmacophore features, comprised of two HBAs, one HBD, and two HYs (Fig. 3).

This pharmacophore model composed of five chemical features selected from the FMO-based hypotheses, and then used as a 3D query to virtual screening of our in-house natural product database, and yielded early 142 virtual hits that match well the optimal pharmacophore features. The compounds were then filtered by fit value (fit value > 1) and chemical diversity, resulting in the selection of 91 natural products for further evaluation.

### Docking study of virtual hits

**Figure 4:**
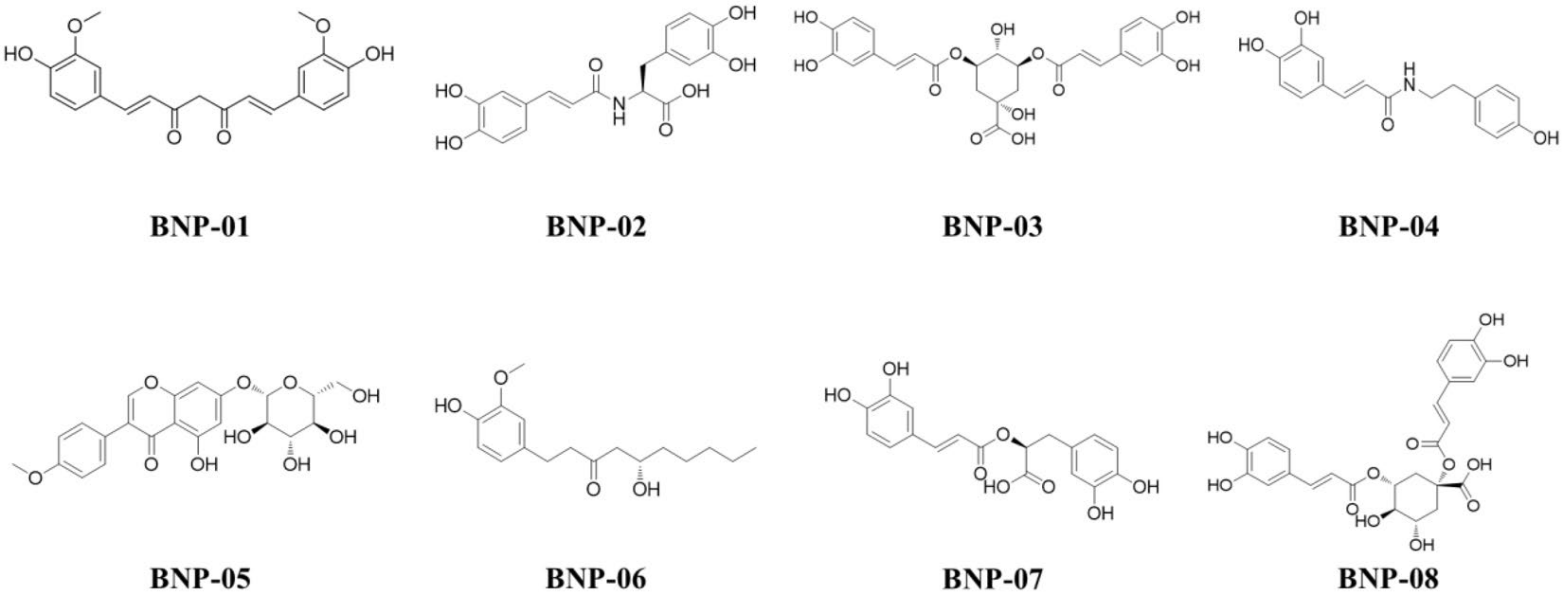
Chemical structures of eight virtual hits selected by the pharmacophore model and molecular docking.

Molecular docking was performed to evaluate the binding potential of the 91 selected natural products. After applying the LigScore1^36^ scoring function (LigScore1 > 2) for filtering, 38 compounds were selected based on exhibiting good interactions with hotspot residues (Asn159, Gln160, Lys194, and Glu196) in PrP^C^ binding site. After integrating the information from the FMO results, the scaffold shape, and predicted binding conformations of the docked compounds, we finally selected the most potent eight candidates among the 38 hit compounds (Supplementary Table S2). The chemical structures of these eight compounds that docked well into the hotspot binding site of PrP^C^ are shown in Fig. 4.

### Inhibition of PrPSc formation in M2B cells with virtual hits

**Figure 5:**
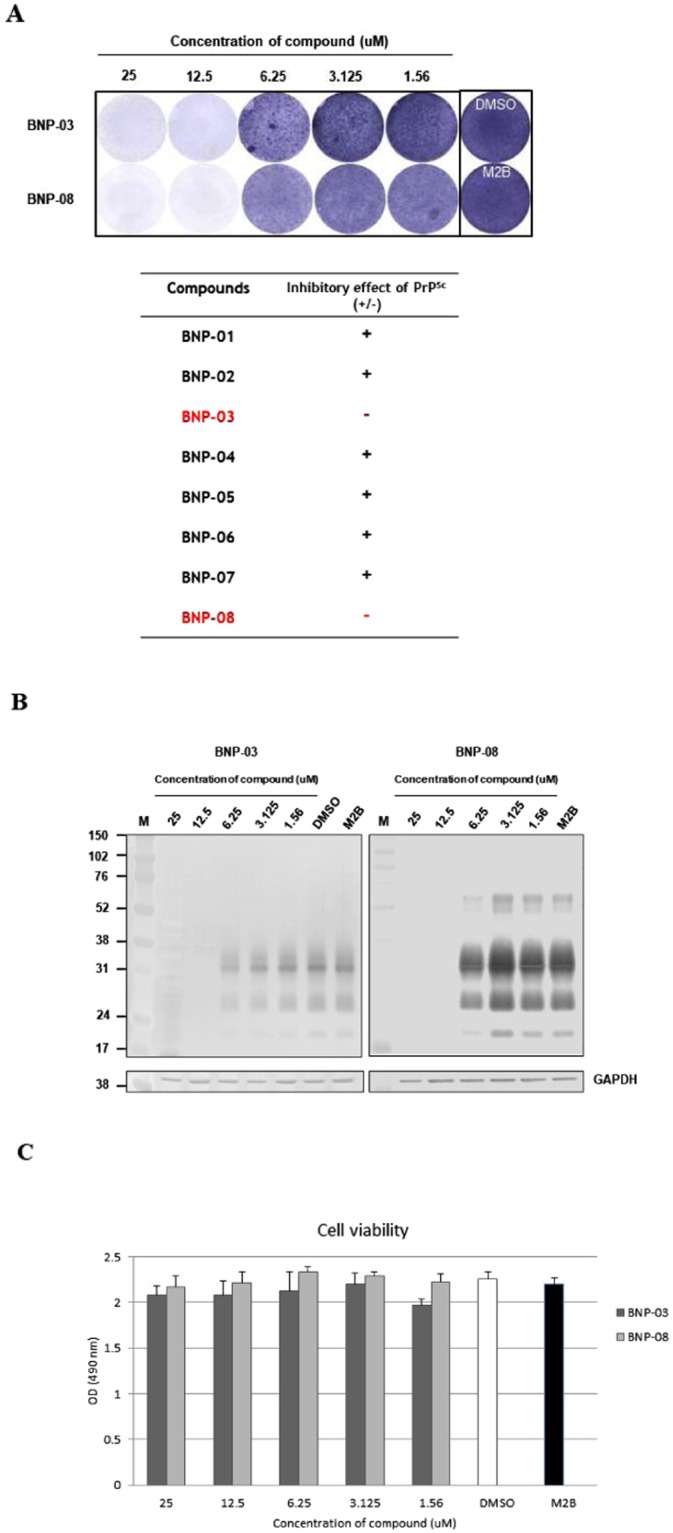
Antiprion activity of chemical compounds in M2B cells. **(A)** Efficiency of PrP^Sc^ clearance for two antiprion compounds was estimated by evaluating the signal sensitivity for PrP^Sc^ on SSCA and the other compounds with no inhibitory effects on PrP^Sc^ accumulation. Inhibitory concentration was valued in the range from 25 μM to 1.56 μM by SSCA. The spots were not observed to treat BNP-03 or BNP-08 from 12.5 μM to 25 μM. **(B)** Western blot analysis of M2B cells following treatment with different concentrations of the natural products BNP-03 (left panel) and BNP-08 (right panel). Two natural products were effective against PrP^BSE^ from the concentration of more than 12.5 μM. **(C)** Cell viability was determined in M2B cells in the presence of the natural products BNP-03 or BNP-08. Values represent the mean of three independent experiments and the standard deviation is shown by error bars.

Although several researchers have been searching for antiprion drugs to block the conversion process, there are currently no proven therapeutic agents for BSE. We first used M2B cells (Persistent BSE-infected cell line), an established *in vitro* cell model against PrP^BSE^, to screen the selected eight virtual hits for a potential effect on PrP^Sc^ levels. To evaluate the spectrum of antiprion activity of these compounds, M2B cells were exposed to indicate the concentrations of compounds and passaged six times. The cells were collected, and following PK digestion, PrP^Sc^ levels in the culture were determined. From the above results, we obtained two natural products (BNP-03 and BNP-08), with antiprion activity that would effectively reduce the PrP^Sc^ levels in SSCA (Fig. 5A). The minimum inhibitory concentration was each 12.5 µM. Natural products, including BNP-01, BNP-02, BNP-04, BNP-05, BNP-06, and BNP-07 failed to reduce the PrP^Sc^ accumulation in SSCA. To confirm the inhibitory concentration, the SSCA result was further verified using WB. Both BNP-03 and BNP-08 were observed to clear PrP^BSE^ in M2B cells at a concentration of 12.5 µM (Fig. 5B).

### Cytotoxicity assay

We also investigated the cytotoxic concentration in the range from 25 to 1.56 µM by a serial dilution method. Cytotoxicity, as determined by the MTS assay, showed no significant cell death in either BNP-03 or BNP-08 treatment in M2B cells. The effective antiprion dose, at 25 or 12.5 µM of each of the two natural products, BNP-03 or BNP-08, produced no significant changes in cell viability (Fig. 5C).

### Binding mode analysis for BNP-03 and BNP-08

From the results of the cell-based assay, we confirmed that the PrP^Sc^ accumulation was inhibited by BNP-03 and BNP-08. To confirm the stability of the top ranked binding conformations of PrP^C^-natural product (BNP-03 and BNP-08) complexes from docking simulation, we performed the MD simulations (Supplementary Fig. S1). The root mean square deviation (RMSD) of the PrP^C^ and the two natural products showed small fluctuations, indicating stability of these two complexes during the MD simulations. To reveal the binding affinity of these two natural products, we evaluated the changes in PIEs of BNP-03 and BNP-08 by performing FMO calculations. Comparison of the PIEs of the protein-ligand complexes provided an important list of interactions, which allowed us to test the structure of ligands for strong, weak, missing, or new interactions with PrP^C^.

**Figure 6:**
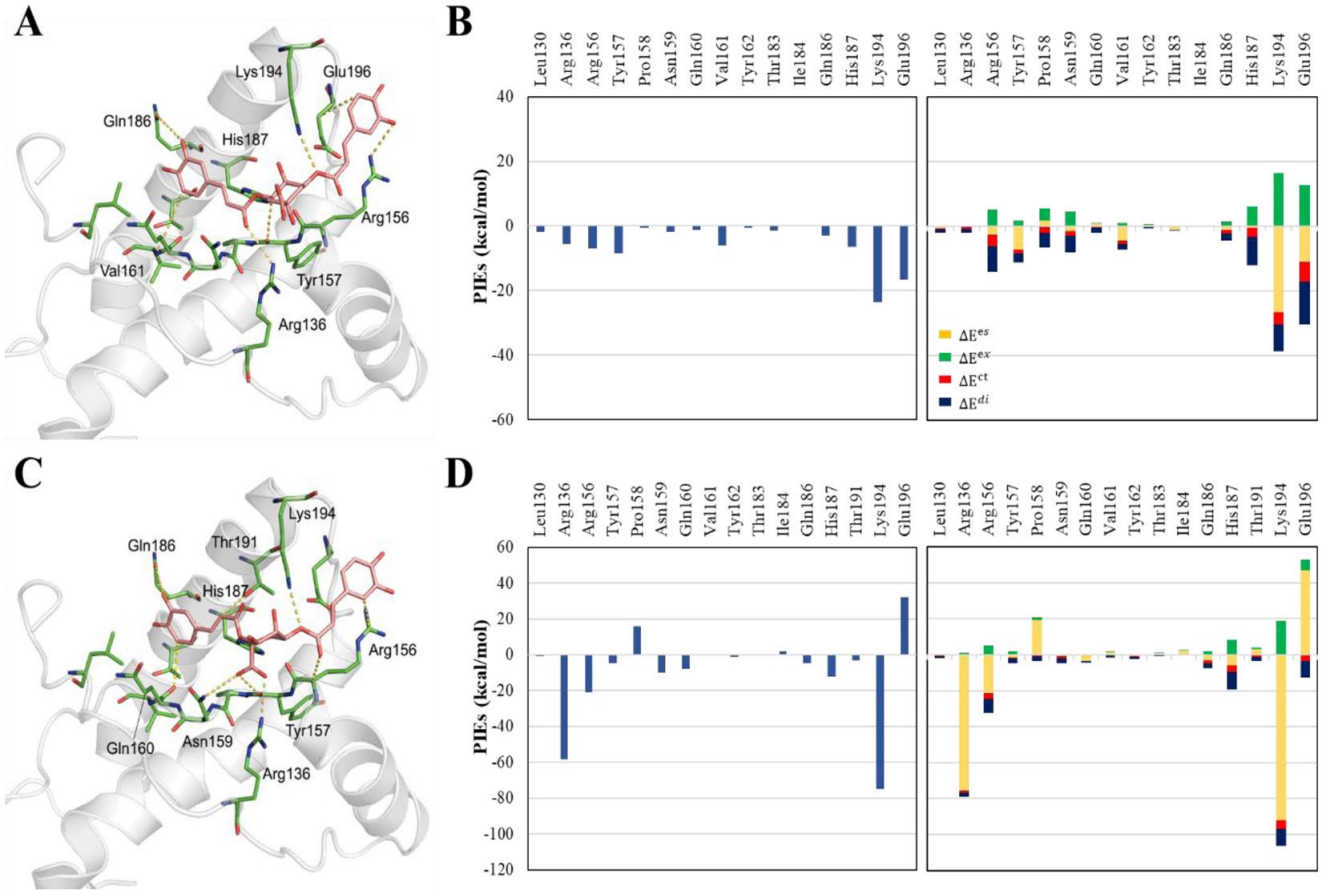
The FMO results for two natural products in complex with PrP^C^. The structure of BNP-03 **(A)** and BNP-08 **(C)** binding to the hotspot binding site of PrP^C^. The ligand is shown in light pink and the protein residues are coloured green. Nitrogen and oxygen atoms are coloured in blue and red, respectively. The key interactions according to the FMO calculations are marked as yellow dashed lines. The left-hand bar plots of BNP-03 **(B)** and BNP-08 **(D)** describe the PIEs of the significant residues in the binding site of PrP^C^, and the light-hand bar plots describe the PIEDA of these key interactions. The electrostatic, exchange repulsion, charge transfer and dispersion terms are coloured in yellow, green, red, and dark blue, respectively.

The PIEs obtained from the FMO calculations of the docked PrP^C^-natural product (BNP-03 and BNP-08) complexes are shown in Fig. 6. The FMO calculations detected six strong interactions between these two natural products and PrP^C^ with six residues: Arg136, Arg156, Tyr157, Gln186, His187, and Lys194 (Fig. 6 and Supplementary Tables S3–S4). As expected, the FMO calculations of the PrP^C^-natural products were mostly similar to the previously detected important interactions of the PrP^C^-GN8 complex (Fig. 2). One of the notable differences was that the PIEs calculated between Lys194 and two natural products were much stronger than those obtained between Lys194 and GN8 by forming hydrogen bond interactions. Apart from the strong interaction with Lys194, the carboxylic group of BNP-08 formed another significant hydrogen bond interaction with Arg136 (Fig. 6C).

To reveal the nature of some important interactions of PrP^C^-natural product complexes, we examined these complex structures separately. In the case of BNP-03, it interacted strongly with Val161 and Glu196 by forming hydrogen bonding interactions, as well as a CH-π interaction through its benzyl ring (Fig. 6A,B). The calculated PIEs of BNP-08 also revealed that it formed a novel strong interaction with Thr191 (Fig. 6C,D). The total PIE of PrP^C^-BNP-03 complex (−86.96 kcal/mol) and PrP^C^-BNP-08 complex (−111.17 kcal/mol) were much stronger than the total PIE of PrP^C^-GN8 complex (−44.66 kcal/mol), indicating that these two compounds could serve as potent drug leads against PrP^C^. Therefore, the FMO method can be used as a useful tool to explore the key interactions that are important in ligand binding. This approach may be highly useful to guide scaffold hopping of ligands to enhance their binding affinity to PrP^C^ in future studies.

## Disscussion

This study included a novel application of the FMO method, which enables the *ab initio* quantum-mechanical calculations of protein-ligand complexes to analyse the interaction of protein and its ligands in structure-based drug design. Through the present study, the FMO-based analysis of interactions between GN8 and PrP^C^ provided important structural features to generate a new pharmacophore model of PrP^C^. We accordingly used this pharmacophore model to virtually screen our in-house natural product library, and performed molecular docking to discover novel potent antiprion compounds. We identified the inhibitory and cytotoxicity effects of virtual hits against PrP^BSE^ in a BSE infected cell-based assay system, and finally selected two novel natural products, BNP-03 and BNP-08. The interactions of the PrP^C^-natural product complexes were then quantitatively studied by using the FMO method at correlated MP2/6-31 G**/PCM level. These data allowed us to generate the interaction patterns between PrP^C^ and ligands and to provide important information to predict the potent antiprion compounds in drug discovery. Overall, the outcome of this study will be used as a promising strategy for searching novel antiprion compounds, and further animal experiments will be performed to confirm the effect of these compounds *in vivo*.

## Methods

### FMO-based virtual screening

In our previous study, the 3D pharmacophore model was generated with computationally derived PrP^c^-GN8 complex from molecular docking^24^. We used the FMO method to analyse the interaction energies between PrP^c^ and GN8, a known antiprion agent to identify important amino acid residues for ligand binding. A new modified 3D pharmacophore model was developed with newly defined pharmacophore features based on the FMO calculation. This pharmacophore model consisted of two hydrophobic groups (HY), two hydrogen-bond acceptors (HBA), and one hydrogen-bond donor (HBD). Then, this modified pharmacophore model was used as a new 3D query for pharmacophore-based virtual screening.

An in-house natural product database, comprising approximately 2,000 compounds with structural diversity was screened in this study. Prior to virtual screening, the database was curated by adding missing hydrogens, deleting ions, removing duplicate compounds and molecules containing atoms other than C, N, O, S, P, and halogens were excluded. Then, the pharmacophore model composed of five chemical features was used to screen the database to identify virtual hits that matched the pharmacophore model by using BIOVIA Discovery Studio 2017 R2.

### Molecular docking study

Molecular docking studies were performed using the CDOCKER program, which is a grid-based molecular docking method with a CHARMm force field in the BIOVIA Discovery Studio 2017 R2. To determine the binding mode of virtual hits to the hotspot binding site of PrP^C^, the NMR structure of PrP^C^ was retrieved form the Protein Data Bank (PDB ID: 1AG2). From the docked structure of the PrP^C^-GN8 complex, GN8 was located in the hotspot binding site of PrP^C^ with key residues (Asn159, Gln160, Lys194, and Glu196). This was used to define the binding site of PrP^C^. All of the ligand binding poses were generated, and the results were scored by the LigScore1 scoring function in BIOVIA Discovery Studio 2017 R2. LigScore1^36^ is one of the scoring functions that accurately predict the binding affinity between ligand and protein. The predicted structures of the protein-ligand complexes were visualised using the PyMOL program (https://www.pymol.org/).

### Treatment of M2B cells with virtual hits

An M2B cell with persistent C-BSE has been established in a previous study^28^. The cells were grown in DMEM containing 10% FBS, antibiotics (penicillin and streptomycin), and the non-essential amino acid, L-glutamine. The compounds were dissolved in DMSO, diluted to produce a 10 mM stock solution, and stored at −20 °C. The compound stock solutions (10 mM) were diluted in DMEM and then further applied to the cells at final concentrations of 1.56, 3.125, 6.25, 12.5, or 25 µM. Control cell cultures were treated with DMSO only (0.1% v/v). Approximately 5,000 M2B cells were seeded in each well (in triplicate) of 96-well plate prior to the addition of compounds. The following day, we added 50 µl of the diluted compounds in the range from 25 to 1.56 µM to each well on every passage. The cells were incubated for 3 days and passaged six times.

### SSCA

The SSCA was performed as described previously^29^. Briefly, ELISPOT plates (MSIPS4510, Millipore, Billerica, MA, USA) were activated using 70% ethanol for 2 min. Twenty-five thousand cells were seeded onto each plate, washed twice, and dried. The dried cells on the membranes were then treated with 2 µg/ml proteinase K in lysis buffer containing 150 mM NaCl, 0.5% sodium deoxycholate and 0.5% Triton X-100 in 50 mM Tris-HCl (pH 8.0) and incubated at 37 °C for 30 min. The plates were washed twice with PBS and then incubated with 1 mM phenylmethylsulfonyl fluoride (PMSF) for 10 min. Plates were then incubated with 3 M guanidium thiocyanate (GSCN). The denatured PrP^Sc^ proteins were detected with the primary antibody, 6H4 (1 mg/ml, 1:4000) and secondary antibody, alkaline phosphatase-conjugated anti-mouse IgG (1 mg/ml, 1:2000, KPL, USA) and detected with alkaline phosphatase conjugate substrate, BCIP/NBT (KPL). The immunostained spots were visualised with CTL ELISPOT equipment (Cellular Technology, Ltd, Shaker Heights, OH).

### Western blotting (WB)

Following treatment with the virtual hits, the cells were lysed using 0.5% Triton X-100, 0.5% sodium deoxycholate, 150 mM NaCl, and 5 mM EDTA in 10 mM Tris-HCl [pH 7.5], and then incubated with 20 µg/ml proteinase K for 20 min at 37 °C. Next, 1 mM 4-(2-Aminoethyl)-benzenesulfonyl fluoreide (Pefabloc, Roche, USA) was added on ice for 5 min to stop PK activity. The proteinase K-treated proteins were centrifuged at 20,000 × *g* for 45 min at 4 °C, and the pelleted protein was resuspended in 25 µg 1 × sample loading buffer (Invitrogen). The electrophoresis was conducted at 200 V for 35 minutes using a pre-casting NuPAGE 12% Bis-Tris gel (invitrogen). Proteins were transferred to a polyvinylidene difluoride (PVDF) membrane (Millipore, USA) and subjected to reaction by rabbit anti-PrP polyAb S1 (produced by Animal and Plant Quarantine Agency, Korea) or GAPDH (glyceraldehydes-3-phosphate dehydrogenase; Abcam, Cambridge, UK) as a control to confirm protein content of cell extracts. The expression of prion protein was detected by LAS 4000 (Fuji, Japan) using CDP star (Applied Biosystem, USA) with chemiluminescence as a color developer.

### Cytotoxicity assay

To test the cytotoxicity of treatment concentrations for each compound, an MTS-based assay, the CellTiter 96^®^ “Aqueous One Solution Cell Proliferation Assay” (Promega, Madison, WI, USA) was employed. The procedures were performed following the manufacturer’s instructions. After treatment with the compounds at five different concentrations between 1.56 and 25 µM or control, M2B cells were plated into 96-well plates in triplicate at a density of 1 × 10^4^ cells per well. To stop the reaction, 25 µl of 10% SDS was added to each well. The plate was measured at 490 nm in an ELISA reader (Beckman, USA).

### MD simulations

The MD simulations of the PrP^C^-natural product (BNP-03 and BNP-08) complexes were performed using the Desmond 4.1 suite implemented in the Maestro program of Schrödinger (Schrödinger, LLC, New York, NY, 2017). Each of these two complexes was placed in an orthorhombic box, which was then filled with water molecules (TIP3P model), and the OPL3 force field was used. In the MD simulation, ions (Na^+^ and Cl^−^) were added to simulate the physiological concentration of monovalent ions (0.15 M). NPT (constant number of particles, pressure, and temperature) with a constant temperature (300 K) and pressure (1.01325 bar) was employed as an ensemble class. The particle-mesh Ewald method^37^ was applied to calculate long-range electrostatic interactions, and the cutoff for van der Waals and short-range electrostatic interactions was truncated to 9 Å. Nosé–Hoover thermostats^38^ were utilized to keep a constant simulation temperature, and the Martina–Tobias–Klein method^39^ was used to control the pressure. A RESPA integrator^40^ was employed to integrate the equations of motion with an inner time step of 2 fs for bonded and non-bonded interactions within the short-range cutoff. The default protocol in Desmond was used for the system to reach equilibrium. After the system reached equilibrium, the MD simulation was performed for a period of 50 ns, and snapshot configurations were saved at each 4 ps interval. Analyses were performed using the root mean square deviation (RMSD) generated from Schrödinger.

### FMO calculations

The FMO calculations of PrP^C^-GN8 and PrP^C^-natural products (BNP-03 and BNP-08) complexes from the top ranked docking conformations were performed with the General Atomic and Molecular Electronic Structure System (GAMESS)^41^, and an energy decomposition analysis was performed at the second-order Moller Plesset (MP2)^42^ level with the 6–31 G** basis set. The PIE (${\triangle {\rm{E}}}_{{\rm{ij}}}^{{\rm{int}}}$) between fragment *i* and fragment *j* consisted of four or five energy terms, depending on whether or not the implicit solvation model was considered: electrostatic (${\triangle {\rm{E}}}^{{\rm{es}}}$), exchange repulsion (${\triangle {\rm{E}}}^{{\rm{ex}}}$), charge transfer (${\triangle {\rm{E}}}^{{ct}}$), dispersion (${\triangle {\rm{E}}}^{{\rm{di}}}$), and solvation energy ($\triangle {{\rm{G}}}_{{\rm{sol}}}$). The PIEs in the FMO calculations was defined by Eq. (1)^43^.

1 $${{\rm{\Delta }}E}_{{\rm{ij}}}^{{\rm{int}}}={{\rm{\Delta }}E}_{{\rm{ij}}}^{{\rm{es}}}+{{\rm{\Delta }}E}_{{\rm{ij}}}^{{\rm{ex}}}+{{\rm{\Delta }}E}_{{\rm{ij}}}^{{\rm{ct}}}+{{\rm{\Delta }}E}_{{\rm{ij}}}^{{\rm{di}}}+{{\rm{\Delta }}G}_{{\rm{sol}}}$$

The PIEs and PIEDA contributions of all residues of PrP^C^ with GN8, BNP-03, and BNP-08 are listed in the Supplementary Information.

## Electronic supplementary material

Supplementary Information

GN8, BNP-03, BNP-08
