# Supplementary material for: Application of the fragment molecular orbital method to discover novel natural products for prion disease
Source: Sci Rep. 2018 Aug 30;8:13063. doi: 10.1038/s41598-018-31080-7 (PMC6117342; doi:10.1038/s41598-018-31080-7)
Supplement: Supplementary file 1 — Supplementary Information [file 41598_2018_31080_MOESM1_ESM.docx]

**Application of the fragment molecular orbital method to discover novel natural products for prion disease**

Jiwon Choi ^1, ‡^, Hyo-Jin Kim ^2, ‡^, Xuemei Jin ^3^, Hocheol Lim ^3^, Songmi Kim ^1^, In-Soon Roh ^2^, Hae-Eun Kang^2^, Kyoung Tai No ^1,3,^* and Hyun-Joo Sohn ^2,^*

^1^ Bioinformatics and Molecular Design Research Center (BMDRC), Yonsei University, Seoul 03722, Republic of Korea

^2^ OIE Reference Laboratory for CWD, Foreign Animal Disease Research Division, Animal and Plant Quarantine Agency, Gimcheon, Gyeongsangbukdo 39660, Korea

^3^ Department of Biotechnology, Yonsei University, Seoul 03722, Korea

^‡^ Co-first authors

* Corresponding author:

E-mail: [ktno@bmdrc.org](mailto:ktno@bmdrc.org) (K.T. No), [shonhj@korea.kr](mailto:shonhj@korea.kr) (H. S.).

**Supplementary Information**

**
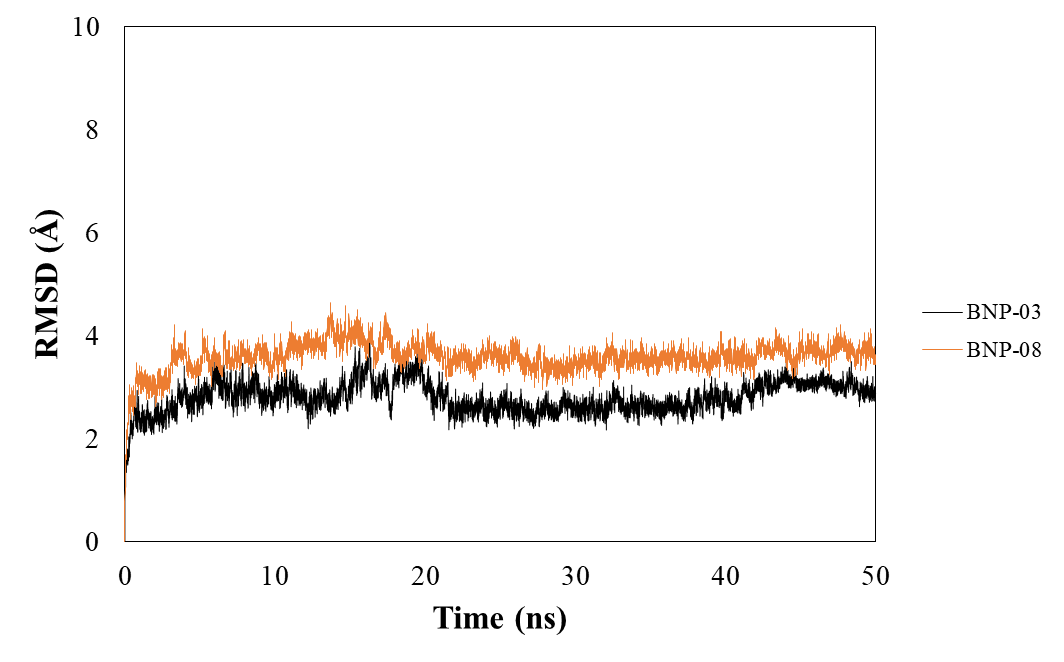
**

**Figure S1**. RMSDs of the PrP^C^-BNP-03 (black) and PrP^C^-BNP-08 (orange) complexes obtained from the 50 ns MD simulations.

**Table S1**. The calculated PIEs (kcal/mol) between GN8 and PrP^C^ in the hotspot binding pocket.

| **Residue** | **PIEs** | ${\boldsymbol{\Delta}\boldsymbol{E}}^{\boldsymbol{es}}$ | ${\boldsymbol{\Delta}\boldsymbol{E}}^{\boldsymbol{ex}}$ | ${\boldsymbol{\Delta}\boldsymbol{E}}^{\boldsymbol{ct}}$ | ${\boldsymbol{\Delta}\boldsymbol{E}}^{\boldsymbol{di}}$ |
| --- | --- | --- | --- | --- | --- |
| Leu130 | 3.518 | -4.316 | 15.241 | -2.356 | -4.859 |
| Arg136 | -7.086 | 0.051 | 4.69 | -1.648 | -2.571 |
| Arg156 | -40.225 | -32.591 | 18.049 | -6.344 | -13.701 |
| Tyr157 | -7.141 | -4.058 | 1.37 | -0.967 | -3.519 |
| Pro158 | -8.398 | -10.269 | 14.376 | -5.159 | -8.189 |
| Asn159 | -20.084 | -19.12 | 13.344 | -1.548 | -11.703 |
| Gln160 | -14.644 | -16.6 | 10.192 | -3.168 | -6.694 |
| Val161 | 11.61 | 11.35 | 7.187 | -1.716 | -5.849 |
| Tyr162 | -2.424 | -2.24 | 2.112 | 0.488 | -2.866 |
| Thr183 | 2.511 | -4.017 | 12.374 | -2.259 | -4.106 |
| Ile184 | -1.013 | -1.829 | 2.439 | -0.618 | -1.725 |
| Gln186 | -1.001 | -0.729 | 0.872 | -0.336 | -1.596 |
| His187 | -5.106 | -1.993 | 6.262 | -2.534 | -7.585 |
| Lys194 | -6.023 | -15.197 | 43.604 | -14.128 | -7.36 |
| Glu196 | 27.659 | 18.666 | 22.705 | -8.867 | -14.642 |

**Table S2**. The LigScore1, CDOCKER energy (kcal/mol), and fit value for the final selected eight compounds and GN8 (no fit value).

| **Name** | **LigSocre 1** | **CDOCKER energy** | **Fit value** |
| --- | --- | --- | --- |
| BNP-01 | 2.67 | -29.532 | 2.694 |
| BNP-02 | 5.16 | -43.791 | 1.954 |
| BNP-03 | 5.85 | -32.185 | 1.039 |
| BNP-04 | 4.87 | -34.743 | 1.18 |
| BNP-05 | 5.54 | -8.336 | 1.346 |
| BNP-06 | 4.54 | -32.044 | 2.984 |
| BNP-07 | 4.77 | -39.020 | 1.887 |
| BNP-08 | 6.13 | -37.278 | 3.679 |
| GN8 | 5.58 | -11.097 |  |

**Table S3**. The calculated PIEs (kcal/mol) between BNP-03 and PrP^C^ in the hotspot binding pocket.

| **Residue** | **PIEs** | ${\boldsymbol{\Delta}\boldsymbol{E}}^{\boldsymbol{es}}$ | ${\boldsymbol{\Delta}\boldsymbol{E}}^{\boldsymbol{ex}}$ | ${\boldsymbol{\Delta}\boldsymbol{E}}^{\boldsymbol{ct}}$ | ${\boldsymbol{\Delta}\boldsymbol{E}}^{\boldsymbol{di}}$ |
| --- | --- | --- | --- | --- | --- |
| Leu130 | -1.978 | -0.615 | 0.18 | -0.379 | -1.052 |
| Arg136 | -5.738 | -0.199 | 0.171 | -0.634 | -1.215 |
| Arg156 | -7.012 | -2.592 | 5.262 | -3.55 | -8.03 |
| Tyr157 | -8.429 | -7.349 | 1.689 | -1.037 | -2.733 |
| Pro158 | -0.601 | 1.702 | 3.777 | -2.002 | -4.569 |
| Asn159 | -1.938 | -1.674 | 4.565 | -1.28 | -5.108 |
| Gln160 | -1.118 | 0.906 | 0.195 | -0.49 | -1.539 |
| Val161 | -6.062 | -4.398 | 0.981 | -1.172 | -1.795 |
| Tyr162 | -0.541 | 0.498 | 0.028 | -0.17 | -0.468 |
| Thr183 | -1.359 | -1.085 | 0.004 | -0.113 | -0.115 |
| Ile184 | 0.24 | -0.068 | -0.001 | -0.044 | 0.018 |
| Gln186 | -3.005 | -1.059 | 1.478 | -1.214 | -2.205 |
| His187 | -6.431 | -0.563 | 6.077 | -2.745 | -8.7 |
| Lys194 | -23.638 | -26.612 | 16.361 | -3.736 | -8.207 |
| Glu196 | -16.587 | -10.934 | 12.605 | -6.187 | -13.255 |

**Table S4**. The calculated PIEs (kcal/mol) between BNP-08 and PrP^C^ in the hotspot binding pocket.

| **Residue** | **PIEs** | ${\boldsymbol{\Delta}\boldsymbol{E}}^{\boldsymbol{es}}$ | ${\boldsymbol{\Delta}\boldsymbol{E}}^{\boldsymbol{ex}}$ | ${\boldsymbol{\Delta}\boldsymbol{E}}^{\boldsymbol{ct}}$ | ${\boldsymbol{\Delta}\boldsymbol{E}}^{\boldsymbol{di}}$ |
| --- | --- | --- | --- | --- | --- |
| Leu130 | -0.776 | -0.059 | 0.406 | -0.666 | -1.427 |
| Arg136 | -58.359 | -75.312 | 1.335 | -1.572 | -2.165 |
| Arg156 | -20.904 | -21.445 | 5.344 | -2.99 | -7.976 |
| Tyr157 | -4.652 | -1.51 | 1.812 | -0.474 | -2.58 |
| Pro158 | 15.666 | 19.518 | 1.544 | -0.698 | -3.014 |
| Asn159 | -9.799 | -0.807 | 0.391 | -1.246 | -2.498 |
| Gln160 | -8.021 | -3.393 | 0.025 | -0.038 | -0.792 |
| Val161 | -0.045 | 1.564 | 0.508 | -0.464 | -1.26 |
| Tyr162 | -1.241 | -0.737 | 0.224 | -0.415 | -1.022 |
| Thr183 | 0.238 | 0.763 | 0.05 | -0.214 | -0.383 |
| Ile184 | 2.141 | 2.509 | 0.001 | -0.113 | -0.077 |
| Gln186 | -4.901 | -3.044 | 1.886 | -1.669 | -2.914 |
| His187 | -12.212 | -6.02 | 8.293 | -3.594 | -9.836 |
| Thr191 | -3.165 | 3.193 | 0.743 | -1.011 | -2.573 |
| Lys194 | -74.887 | -92.153 | 18.915 | -4.796 | -9.253 |
| Glu196 | 32.009 | 46.917 | 6.055 | -3.455 | -9.212 |
